# Molecular determinants of the DprA−RecA interaction for nucleation on ssDNA

**DOI:** 10.1093/nar/gku349

**Published:** 2014-04-29

**Authors:** Johnny Lisboa, Jessica Andreani, Dyana Sanchez, Marion Boudes, Bruno Collinet, Dominique Liger, Herman van Tilbeurgh, Raphael Guérois, Sophie Quevillon-Cheruel

**Affiliations:** 1 Université Paris-Sud, Institut de Biochimie et de Biophysique Moléculaire et Cellulaire, UMR 8619, F-91405 Orsay, France; 2 CEA, iBiTecS, F-91191 Gif sur Yvette, France; 3 Université Paris-Sud & CNRS, UMR 8221, F-91191 Gif sur Yvette, France; 4 UFR sciences de la vie, Université Pierre et Marie Curie UPMC, Sorbonne Universités, F-75005 Paris, France

## Abstract

Natural transformation is a major mechanism of horizontal gene transfer in bacteria that depends on DNA recombination. RecA is central to the homologous recombination pathway, catalyzing DNA strand invasion and homology search. DprA was shown to be a key binding partner of RecA acting as a specific mediator for its loading on the incoming exogenous ssDNA. Although the 3D structures of both RecA and DprA have been solved, the mechanisms underlying their cross-talk remained elusive. By combining molecular docking simulations and experimental validation, we identified a region on RecA, buried at its self-assembly interface and involving three basic residues that contact an acidic triad of DprA previously shown to be crucial for the interaction. At the core of these patches, ^DprA^M238 and ^RecA^F230 are involved in the interaction. The other DprA binding regions of RecA could involve the N-terminal α-helix and a DNA-binding region. Our data favor a model of DprA acting as a cap of the RecA filament, involving a DprA−RecA interplay at two levels: their own oligomeric states and their respective interaction with DNA. Our model forms the basis for a mechanistic explanation of how DprA can act as a mediator for the loading of RecA on ssDNA.

## INTRODUCTION

Homologous recombination, the process of exchanging genetic material between DNA strands with homologous sequences, is important in bacteria for the repair of damaged DNA and for the generation of genomic diversity. The ubiquitous bacterial recombinase RecA plays a central role in several processes based on homologous genetic recombination: recombination-based DNA repair, induction of the cellular SOS responses, restart of collapsed replication forks and natural genetic transformation. All of these activities require the loading of RecA on ssDNA followed by the formation of a RecA-ATP-ssDNA nucleoprotein filament (1) as visualized by electron microscopy and crystallography (2). According to *in vitro* single molecule studies on *^Ec^*RecA (RecA from *Escherichia coli*), it was established that a dimer of RecA is required for nucleation on ssDNA, followed by growth of the filament through monomer addition. Filament growth *in vitro* is unidirectional in presence of ATP, and bidirectional in presence of ATPγS, albeit faster in the 5′→3′ direction (3,4).

The *^Ec^*RecA monomer folds into three domains (1). The core domain contains the ATP binding site, which faces the interior of the filament with the two disordered Loop1 and Loop2 that are part of the ssDNA binding site (5). The C-terminal domain is located on the outer surface of the filament and is involved in the binding of dsDNA (6). The N-terminal domain folds into an α-helix that packs against the neighboring subunit in the filament. This helix is followed by a long linker connecting it to the core domain. Mutations at positions K6 and R28 of *^Ec^*RecA were shown to disrupt the stability of the DNA-free RecA oligomer, although it did not affect the formation of the active nucleoprotein filament (7).

*In vivo*, RecA activity is tightly regulated so that the dynamic nucleoprotein filament is assembled only where and when homologous recombination is needed. Recombination mediators are required to regulate and facilitate the assembly of RecA onto ssDNA (8). These mediators act by displacing the ssDNA-binding protein (SSB) bound to ssDNA tracts to protect them from degradation. The mediators thereby promote assembly of the RecA filament. In the absence of mediators, SSB inhibits recombination by competing with RecA for DNA-binding sites. Three classes of recombination mediators have been described so far, each dedicated to distinct cellular functions. (i) In the repair of gapped DNA, the RecFOR complex promotes homologous recombination by accelerating the assembly of RecA onto ssDNA initially coated by SSB (9,10). *In vitro*, RecF stimulates RecA nucleation, while the RecOR component further accelerates both RecA nucleation and filament growth (1,11,12). (ii) The repair of double-stranded DNA breaks by homologous recombination is rather initiated by the so-called RecBCD (*E. coli*) helicase–nuclease enzymes. These multisubunit machineries resect the DNA ends and load RecA onto the DNA to initiate homologous recombination (13). (iii) The process of natural transformation in bacteria such as *Streptococcus pneumoniae* (*Sp*) link DprA and RecA. *In vitro* studies using heterologous proteins showed that *^Sp^*DprA lowers the barrier raised by *^Ec^*SSB for *^Ec^*RecA access to ssDNA (14). In *S. pneumoniae* however, incoming DNA is immediately degraded in the absence of DprA. This finding implies that ssDNA requires protection prior to the search for homology and that DprA is needed for this protection (15). It is possible that the incoming ssDNA, coated by DprA, may not be covered by SSB, suggesting that DprA can load RecA directly without the necessity of displacing SSB. In *Bacillus subtilis*, DprA has two distinct activities: to facilitate RecA nucleation and RecA-ssDNA filament extension onto ssDNA coated with SsbB or SsbA, and to mediate ssDNA annealing of complementary strands coated by SsbB (16,17).

In previous work, the interplay between *^Ec^*RecA and *^Sp^*DprA in the natural transformation process for *S. pneumoniae* suggested that an interaction between the heterologous proteins might exist. These studies could not specify the oligomeric states of the proteins, nor whether the presence of ssDNA was necessary for DprA and RecA to interact (14). A functional scheme, focused on the *S. pneumoniae* proteins, was proposed to account for DprA−RecA interaction (18). Three acidic residues important for the DprA−RecA interaction (E235/D243/E265, hereafter named the EDE triad) were identified in *^Sp^*DprA by yeast two-hybrid experiments (Y2H) and were shown to cluster at the surface of the X-ray structure of DprA. The EDE triad is located near the dimerization zone of DprA, on each of the two acidic faces generated by the 2-fold rotational symmetry of the DprA homodimer. By mutating the acidic EDE triad into neutral QNQ, it was established by TAP-tag in *S. pneumoniae* that the RecA−DprA interaction, mediated by the charged triad on DprA, was important for transformation. In addition, it was shown that residues G249/S250/I263/Q264/L269/T271/D275, involved in the DprA dimerization zone, could be also engaged in the interaction between DprA and RecA. These experiments suggested that a step involving the rearrangement or disruption of the DprA dimer likely occurs, followed by the nucleation of RecA and its subsequent polymerization onto ssDNA (18).

In the present work, we further probed the nature of the *^Sp^*DprA−*^Sp^*RecA interaction by combining X-ray structure analyses and molecular docking simulations. The docking strategy relied on the structural and evolutionary properties of the docked partners, while imposing the involvement of the EDE triad on DprA (18). The docking strategy used three of the most up-to-date protein–protein docking and scoring techniques (19). The models of interfaces arising from these independent docking simulations were remarkably consistent and helped us to identify the major features of the complex interface. From the predicted models, several mutants could be designed so as to experimentally disrupt the interaction. Using yeast two-hybrid as a reporter system for the interaction, we found that a basic patch on *^Sp^*RecA (the so-called RRK triad) is involved in the interaction and is consistent with the location of the acidic EDE triad on *^Sp^*DprA. In the same region, we also highlighted two central hydrophobic residues, ^DprA^M238 and ^RecA^F230, which likely act as interface anchors and thus play a pivotal role in the interaction. The docking models also suggest that a DNA-binding region of RecA, the so-called Loop2, may bear some role in its interaction with DprA.

As highlighted in the mechanistic model previously proposed in (18), there must be an interplay between the DprA−RecA interaction, the oligomeric state of each partner and their respective interactions with DNA. We thus further probed whether mutations of RecA affecting the interaction between DprA and RecA would also impact RecA self-interaction. We confirmed that the N-terminal α-helix of RecA is a major determinant for its self-interaction and proposed that the ^RecA^F230 residue anchoring the DprA−RecA interaction also plays a central role in RecA oligomerization. By contrast, we found that the RRK triad and the Loop2 contribute to a lesser extent to the oligomerization of RecA. To complete a mechanistic model of DprA as a mediator of RecA in *S. pneumoniae*, we finally mapped the DNA binding site on DprA using multiple mutants of surface residues, and showed that the DNA-binding region of DprA is located opposite to the RecA binding site. All together, these data lead us to propose a structure-based mechanistic model explaining how DprA can release ssDNA and promote the loading of RecA on ssDNA.

## MATERIALS AND METHODS

### Conservation and evolutionary analyses

The coupled alignments for DprA and RecA for InterEvScore were generated using InterEvolAlign server (20), restricting the search to bacterial sequences. These alignments contain 99 species with 35% to 90% mutual sequence identity.

### Input structures for the docking simulations

The 3D structure of DprA from *S. pneumoniae* was used for the docking simulations (PDB ID: 3UQZ) (18). The docking simulations were performed using a dimer of DprA against a monomer of RecA, so we could explore the likelihood of assemblies in which one RecA molecule would bridge between two DprA arranged as in its homodimeric structure.

The structure of *S. pneumoniae* RecA has not been experimentally determined. However, RecA is an extremely well conserved protein and the 3D structure of *^Ec^*RecA was determined by X-ray crystallography at 2.8 Å resolution (2). *^Sp^*RecA monomer was thus obtained by homology modeling starting from this *E. coli* template (PDB ID: 3CMW; 62% sequence identity) and using the SWISS-MODEL web server (2,21). The resulting model is of very high quality with a QMEAN Z-score of 0.017 (22). The RRK basic triad that we further characterized is conserved in both species.

The N-terminal α-helix of RecA, followed by a long flexible region (about 30 residues), is involved in the multimerization of RecA and thus has a very different position in various RecA structures (PDB IDs: 3CMW, 1U94, 3HR8, 2REB). Because the flexibility generated by the long loop between the helix and the rest of the structure cannot be treated, the 50 N-terminal residues of *^Sp^*RecA were removed in the docking simulations. Another flexible region in *^Sp^*RecA (Loop2, residues R209-T221, involved in DNA binding) was removed in the rigid-body sampling and associated scoring simulations. Indeed, this region contains a hydrophobic motif with good sequence conservation (VGVMF) that turned out as rather ‘sticky’ in non-guided rigid-body sampling. On the other hand, the guided docking simulations with High Ambiguity Driven biomolecular DOCKing (HADDOCK) are not hindered by the presence of this loop.

### Modeling by ZDOCK, ZRANK and InterEvScore

ZDOCK 3.0 (23) with dense rotational sampling was used to generate 54 000 rigid-body decoys from the unbound structures of both binding partners. These decoys were subsequently re-scored and re-ranked using ZRANK (version 1) (24) following addition of hydrogens to the structures using Reduce (25). The 54 000 decoys were also re-ranked using InterEvScore (26) with standard options.

The ZRANK and InterEvScore results were clustered using the following parameters: the 1000 decoys with best scores were clustered on the basis of their mutual ligand rmsd values, using the clustering script provided in the InterEvScore package, with the greedy algorithm from (27) with a ligand rmsd cutoff of 7.5 Å. The greedy clustering algorithm involves taking out the structure with the largest number of neighbors, together with these neighbors, as the largest cluster, and then iteratively extracting clusters always starting with the remaining structure that has the largest number of neighbors.

### Modeling by HADDOCK

HADDOCK (version 2.0) (28,29) was used to generate 1000 conformations through rigid-body docking, followed by semi-flexible refinement of the 200 best structures and final refinement in explicit solvent. The 200 resulting refined structures were then clustered on the basis of mutual interface rmsd values (threshold set to 3.0 Å) using the standard HADDOCK algorithm (greedy algorithm from (27)). The clusters were examined on the basis of their size and the scores of the decoys they contained.

HADDOCK takes as input the two structures and a set of ambiguous restraints: active residues are the residues that are known or most strongly believed to be part of the interface, and passive residues are potential interface members (typically, all solvent-accessible neighbors of active residues). We applied standard HADDOCK settings where 50% of the restraints are discarded at random during docking and refinement: this compensates for possible errors in the definition of interface residues, in particular in cases where the active residues are defined from predictions (evolutionary conservation for instance).

For DprA, active residues were defined as the previously identified EDE triad and immediate neighbors, forming a contiguous and well-conserved patch, namely E235, M238, D243, V244, E265, G266, A267 and E281. Passive residues were defined as all solvent-accessible neighbors of active residues, forming a large surface: R217, L231, I232, R236, E239, G241, R242, F245, H261, L262, I263, Q264, K268, E279, F280 and F282. HADDOCK simulations started from a DprA dimer, but they were performed using constraints on a single monomer as there was no evidence that two RecA molecules could bind simultaneously (one on each DprA monomer).

For the main patch on RecA, active residues were defined as the central residues in the most evolutionarily conserved patch: R182, M183, S185, R189, K229, F230, Y231, R235, K265 and K267. Passive residues were defined as the solvent-accessible neighbors of active residues, namely R73, I74, Q186, M188, K190, G192, A193, G225, R226, S233, V264, V268, A269, P270 and P271. For the small conserved patch on RecA, active residues were defined as E335, I336, D337, K338, Q339, D348, G349 and E350, and passive residues were defined as positions E328, F333, D334, V340, R341, L346, I347, E351, V352 and S353.

### Cloning and yeast two-hybrid assays, control of protein expression by western blot

The multiple mutants were obtained by gene synthesis (Genscript) and the single mutants by site-directed mutagenesis using the QuikChange II site directed mutagenesis kit (Novagen). Yeast strain pJ69-4A was the host for Y2H experiments, and pGAD-C1 and pGBDU-C1 were used as the starting material for generating plasmids encoding Gal4-AD and Gal4-BD fusion proteins, respectively (30). cDNA of *S. pneumoniae* RecA (Sp1940) was subcloned into pGAD-C1 as well as into pGBDU-C1 in order to test RecA for self-assembly in Y2H context. Three versions with varying length of the N-terminal α-helix of RecA (full-length FL, Δ28 and Δ50) were constructed. These three variants were the recipients of three mutated areas: F230A, R189A/R226A/K267A (RRK basic triad) and V212A/G213A/V214A/M215A/F216A (hydrophobic stretch on Loop2). cDNA encoding *S. pneumoniae* wild-type DprA (Sp1266) as well as DprA mutants (M238A and E235A/D243A/E265A (EDE triad)) were subcloned into pGBDU-C1. Suitable pairs of pGAD-C1 and pGBDU-C1 variants were introduced into pJ69-4A strain by co-transformation and transformants were selected on complete medium lacking leucine and uracil. Interaction between AD and BD fusions was assayed in selected clones on synthetic complete medium lacking leucine, uracil and histidine. The presence of the RecA fusions in cells was checked by western blot on total/soluble yeast extracts using antibodies raised against Gal4-AD (monoclonal antibodies from Clontech).

### Characterization of the direct protein–protein and protein–ssDNA interactions

To overproduce in *E. coli* the various DprA (WT, QNQ, 5KA, 3KA, 2KA, R115A, R115A/3KA) and ^Δ28^RecA proteins, the genes were amplified by polymerase chain reaction using the templates developed for the Y2H or mutated as described in SI Materials and Methods. The proteins were purified from *E. coli* by a two-step procedure, i.e. a Ni-NTA column followed by a gel filtration. Details regarding these protocols are given in SI Materials and Methods section. Biophysical characterization was performed by SEC-MALS, and the direct interaction between DprA and RecA or between DprA and ssDNA was tested by pull-down experiments on magnetic beads and spectrofluorimetry (see Supplementary Materials and Methods for details).

## RESULTS

### Structural and evolutionary analysis of DprA and RecA

**Figure 1.**
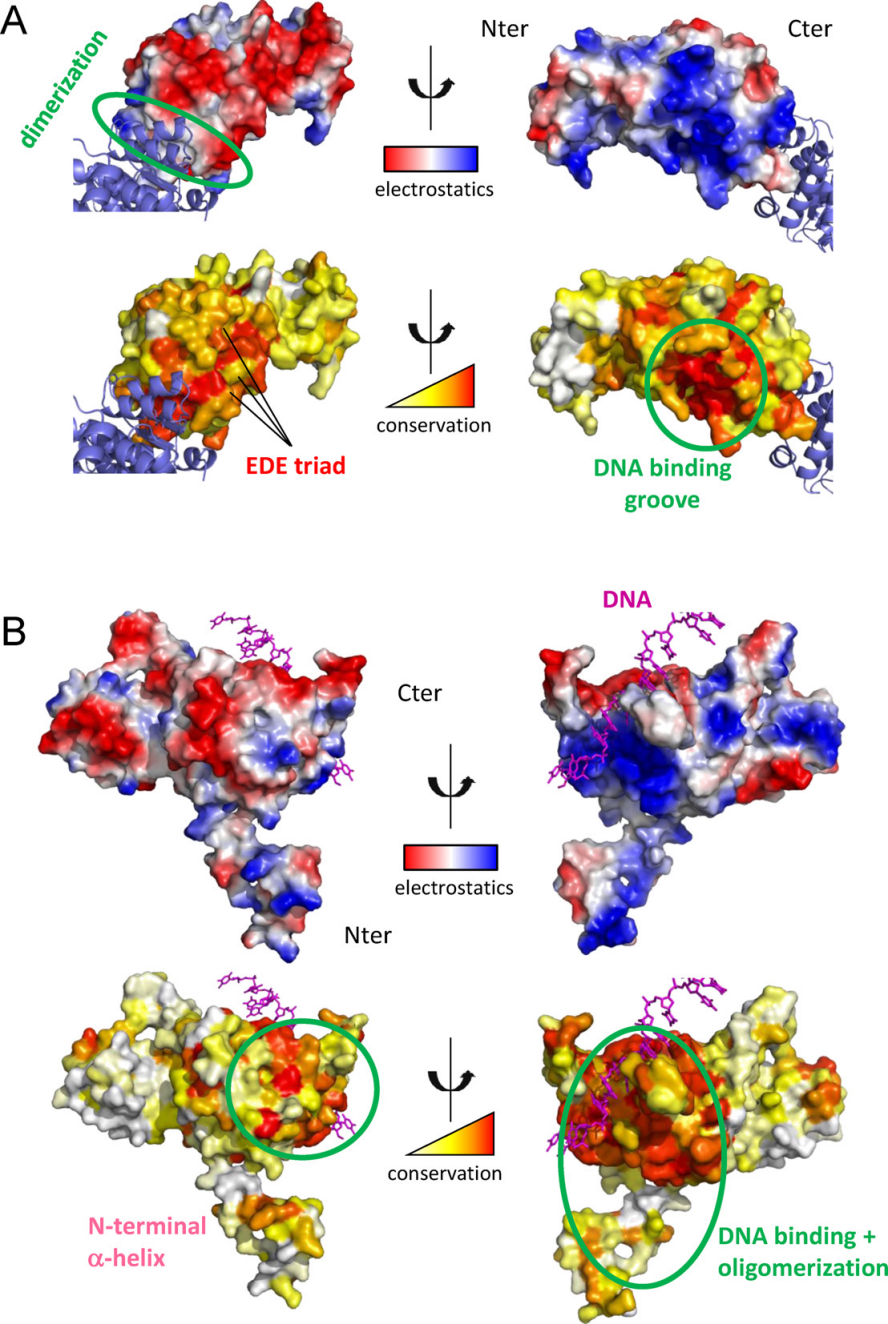
Structural and evolutionary analysis of the binding partners. Electrostatic surfaces were generated using PyMol (The PyMOL Molecular Graphics System, Version 1.5.0.4 Schrödinger, LLC.) (**A**) *^Sp^*DprA surface electrostatics (top) and relative evolutionary conservation (bottom). Positively charged regions are in blue and negatively ones in red. The evolutionary conservation scale is colored from white (poorly conserved) to red (highly conserved). The second DprA monomer represented in blue ribbon highlights the dimerization interface that is circled in green. The EDE triad identified in (18) is marked on one monomer. The putative DNA binding groove, characterized by high conservation and highly positive surface, is circled in green. (**B**) *^Ec^*RecA surface electrostatics (top) and relative evolutionary conservation (bottom). Single-strand DNA is represented in magenta. The DNA binding groove and the multimerization regions are circled in green.

As we initially had no information on the regions of RecA that interacted with DprA, we first explored whether evolutionary constraints could help us to decipher potential binding sites. Both DprA and RecA proteins are found in a number of bacterial species and multiple sequence alignments containing 99 diverse sequences could be derived for both DprA and RecA partners using the InterEvolAlign server (20). From these alignments, the relative evolutionary conservation could be analyzed using the Rate4Site algorithm (31) and mapped onto the surface of both protein structures (Figure 1).

Two regions of DprA are extremely well conserved within the bacterial kingdom. One of them corresponds to the dimerization zone that is electrostatically neutral (Figure 1A). The second conserved patch corresponds to a highly basic surface most probably featuring the DNA binding region. On the opposite side, a third region is also significantly conserved, but less than the first two, exhibiting a highly acidic character and containing the E235/D243/E265 triad (EDE triad) whose mutation was previously shown to weaken the interaction with RecA (18). Interestingly, the overall acidic character of this region is conserved but the precise position of the acidic residues shuffled during evolution (Supplementary Figure S1). So far, the evidence for a direct physical interaction between *^Sp^*DprA and *^Sp^*RecA, independently of any other factors, had not been established. Evidence for the interaction between *^Sp^*DprA and *^Sp^*RecA has been provided by TAP-tag (18), Y2H and *in vitro*, only in presence of ssDNA by electronic microscopy (14). Thus, we first performed biochemical characterization of the direct interaction between the two proteins by pull-down on magnetic beads using purified proteins (Supplementary Figure S2). ^WT^DprA, ^5KA^DprA and the ^AR^DprA monomer (described in (18)) bind specifically to ^Δ28^RecA. The interaction of the 5KA and AR mutants seems stronger compared to WT, which might be due to a lower electrostatic repulsion. ^QNQ^DprA is the most significantly affected mutant with a loss of more than 40% of its apparent affinity for RecA, in very good agreement with the two-hybrid results.

Concerning RecA, the most conserved regions are those containing the DNA binding groove and two surfaces involved in oligomerization (Figure 1B). Apart from these regions, only a very small conserved patch is found on the RecA surface. The regions of RecA that have both high conservation and good electrostatic complementarity with the EDE triad on DprA are thus (i) the multimerization zone on the positive side of RecA and (ii) the small conserved patch.

### Principles of the molecular docking strategy

To further explore the potential binding modes between DprA and RecA, three different docking simulations approaches were used, each relying on specific features for the docking and the scoring of predicted structural assemblies. Two of them (ZRANK (24) and InterEvScore (26)) rely on scoring functions which are based on a rigid-body systematic exploration of all combinations of potential binding modes (rigid-body docking performed by ZDOCK (32)). The third approach (HADDOCK (28)) allows flexibility but requires to restrict the search to one potential binding site on each partner. Between the two rigid-body scoring approaches, ZRANK is mainly driven by physico-chemical constraints while InterEvScore, our in-house developed approach, relies on a knowledge-based, multi-body statistical potential coupled to evolutionary information. The convergence of these three independent approaches on models that are consistent with one another, even though the docking methods are based on very different underlying principles, is thus a good indicator of the reliability of the obtained solution (33).

### Docking with ZDOCK, ZRANK and InterEvScore

Rigid-body sampling was performed with ZDOCK to dock the *^Sp^*RecA monomer (modeled from the *^Ec^*RecA structure, and after removal of the flexible regions) against the *^Sp^*DprA dimer. At this stage, we did not apply any prior constraint forcing the EDE triad to be part of the interface, since we could then control the reliability of the blind docking procedure in its ability to retrieve plausible solutions. The 54 000 decoys generated by ZDOCK were re-scored and re-ranked using ZRANK and InterEvScore. The solutions were clustered for each scoring function on the basis of their mutual ligand rmsd, and only then we considered which models among the obtained solutions satisfied the constraint of the EDE triad in the DprA interface composition.

With ZRANK, the second largest cluster that includes the 13th best score out of 54 000 decoys, contained solutions in which the EDE triad is at the complex interface. This solution, hereafter named ‘best ZRANK model’, involves the large conserved oligomerization patch as the interacting region on RecA (data not shown).

With InterEvScore, among the 10 solutions with best scores, four involve the DprA EDE triad. These four decoys correspond to the 1st, 2nd, 3rd and 8th best scores and are clustered together. Moreover, the interface in these structures also involves the large conserved oligomerization patch identified on RecA. The consensus interface from this cluster thus seems to be a plausible interface with respect to available information about the interface. The model with the best InterEvScore energy among all 54 000 decoys is hereafter named ‘best InterEvScore model’.

### Docking under constraints with HADDOCK

The guided docking step is an independent and complementary approach where we can make full use of the known DprA mutants that disrupt the interaction with RecA. In this step, we performed simulations using restraints, starting as above from a RecA monomer and a DprA dimer. The spatial ambiguous restraints used in all HADDOCK simulations involved: on DprA, the known EDE triad disrupting interaction with RecA; and on RecA, either the oligomerization region with high conservation and basic electrostatic profile or the small conserved patch involving basic residues. We quickly found that no likely cluster of solutions was obtained with the small conserved patch and we thus concentrated on the oligomerization region in the rest of our work.

Two large clusters of solutions were found in these HADDOCK simulations, which correspond to different relative orientations of DprA and RecA. Both clusters contained solutions well ranked by HADDOCK (Supplementary Figure S3). Strikingly, the largest cluster (Cluster 1) contained models with a relative orientation of DprA and RecA that was very close to the best InterEvScore model pinpointed above (interface backbone rmsd between the best InterEvScore model and the representative model of HADDOCK Cluster 1: 2.6 Å). The HADDOCK model that is representative of Cluster 1 is hereafter called ‘best HADDOCK/InterEvScore model’. On the contrary, no solution close to the best ZRANK model was singled out by HADDOCK, and no solution close to the second largest HADDOCK cluster (Cluster 2) was well ranked by either ZRANK or InterEvScore. Therefore, the best consensus from the docking simulations is the solution favored both by InterEvScore and HADDOCK. It is worth noting that all solutions singled out above (the best solutions in the HADDOCK and InterEvScore simulations, the best ZRANK model and the second-best HADDOCK model) involve similar regions on DprA and RecA, only with different relative orientations of the two binding partners.

### Molecular determinants for the DprA−RecA interaction from the best consensus model

**Figure 2.**
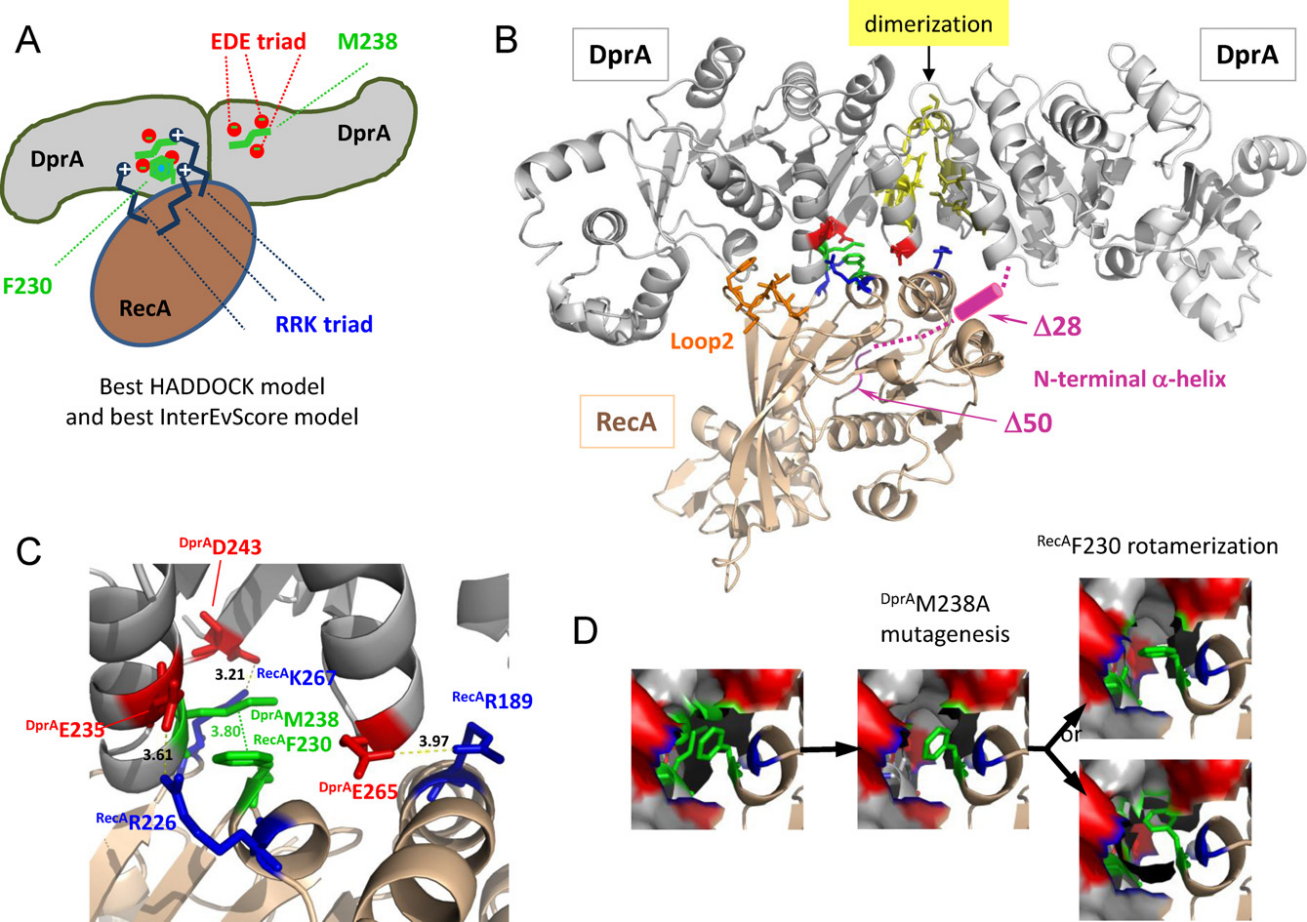
Docking simulation results. (**A**) Schematic representation of the favored HADDOCK model from the docking runs (close to the best InterEvScore model). The central ^RecA^F230, also involved in RecA multimerization, makes apolar contacts with a patch involving ^DprA^M238. The ^DprA^EDE triad is in contact with the RRK triad of RecA. (**B**) Best HADDOCK/InterEvScore model. Ribbon schematic representation of the DprA−RecA complex. The dimer of DprA is in gray and the monomer of RecA is in golden yellow. The residues highlighted in this work are shown in sticks and colored: ^DprA^M238 and ^RecA^F230 are in green, the EDE triad of DprA is in red, the RRK triad of RecA is in blue, the hydrophobic stretch of ^RecA^Loop2 is in orange; the N-terminal α-helix of RecA is in pink and probably points behind the DprA dimer. The dimerization zone of DprA is in yellow. (**C**) The DprA−RecA interaction zone is enlarged to show the residues (represented using the same color code as in (A)) involved in the interaction. Hydrogen bonds and salt bridges are represented by dashed lines, and distances between residues are indicated in Å. The main anchor in this interface is ^RecA^F230, in apolar contact with ^DprA^M238. (**D**) Possible molecular interpretation of the fact that the ^DprA^M238A mutant displays reinforced interaction with ^Δ28^RecA and ^Δ50^RecA compared to ^WT^DprA and loses interaction with ^F230A^RecA. Upon ^DprA^M238A mutation, a pocket would be created in the interface where the ^RecA^F230 might relocate in a more favorable rotameric state.

We focus consequently on the favored consensus model of the interaction between DprA and RecA: the convergent best HADDOCK/InterEvScore model is schematized in Figure 2A and is shown in Figure 2B. This model involves the following set of interface residues: the DprA EDE acidic triad,^ DprA^M238, three basic residues R189/R226/K267 on RecA (the basic RRK triad) and ^RecA^F230. In the best HADDOCK/InterEvScore model, the central ^RecA^F230 is an interface anchor (i.e. it buries a large amount of exposed surface upon binding) and it makes apolar contacts with an apolar patch involving ^DprA^M238, and the EDE triad on DprA is in contact with the basic RRK triad on RecA (Figure 2C). In the proposed DprA–RecA complex model, most of the binding interface arises from the binding of one monomer of RecA against one DprA monomer. The binding of a second RecA molecule to the other DprA monomer would not be possible due to drastic steric clashes, supporting a 2:1 stoichiometry for the initial DprA–RecA complex.

This model and the analysis of the evolutionary conserved regions on RecA also suggest a possible role for two additional flexible regions on RecA: the N-terminal α-helix [M1-Q50], already known to be extensively involved in the oligomerization interfaces of RecA, and a well-conserved hydrophobic stretch of ^RecA^Loop2 [V212/G213/V214/M215/F216] involved in DNA binding. These two regions were not considered in any of the docking simulations because of their high flexibility in a RecA monomeric context, intractable with current docking techniques. The N-terminal α-helix is connected to the core domain by a long and very flexible linker, and this helix may likely fold back onto DprA to form a second interface region. The hydrophobic stretch within Loop2 is close to the interface suggested by the docking models and might also contribute to the binding with DprA.

We used the yeast two-hybrid system to experimentally challenge the implication of the residues considered for the interaction: the N-terminal α-helix of RecA [M1-Q50], the basic patch of RecA (R189/R226/K267) opposite to the acidic one of DprA (E235/D243/E265 triad), the ^DprA^M238 opposite to the ^RecA^F230, and the hydrophobic region of ^RecA^Loop2. Expression and stability of RecA variants fused to Gal4-AD were confirmed by western blotting on yeast total and/or soluble extracts (Supplementary Figure S4).

### A dual role for the N-terminal α-helix of RecA in self-interaction and in the interaction with DprA

**Figure 3.**
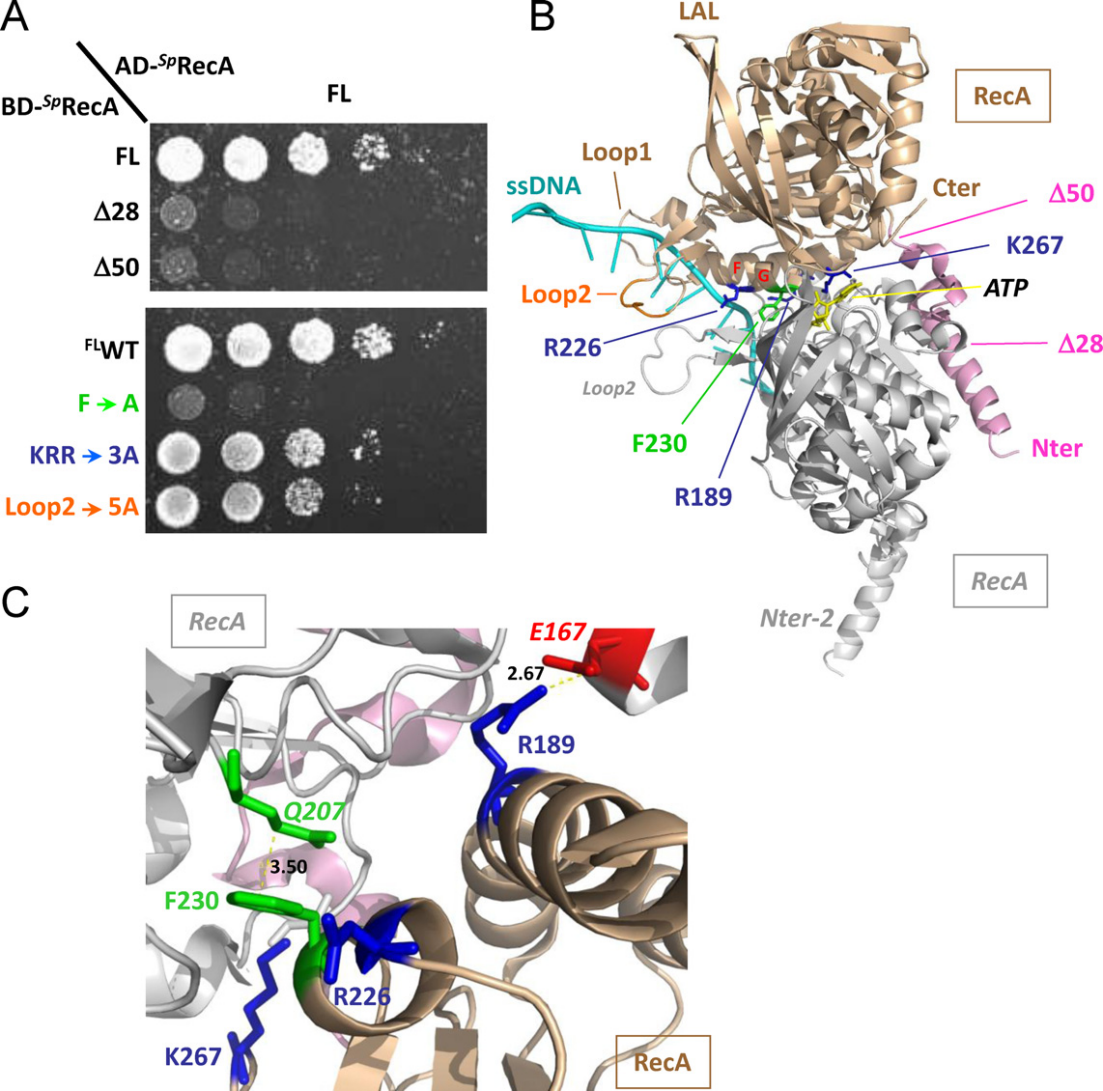
RecA−RecA interaction. (**A**) Yeast strains expressing wild-type *^Sp^*RecA fused to Gal4 activation domain (AD-*^Sp^*RecA) and *^Sp^*RecA variants as Gal4 binding domain fusions (BD-*^Sp^*RecA) were spotted as a series of 1/5th dilutions on selective medium lacking histidine. Plates were incubated for 5 days at 28°C. (**B**) Ribbon schematic representation of the *^Sp^*RecA−*^Sp^*RecA dimer modeled from the X-ray structure of *^Ec^*RecA−ssDNA nucleo-filament (PDB ID: 3CMW (2)). The two monomers are in gold and gray, the ssDNA is in cyan. The color code for the residues concerned in the DprA interaction is the same as in Figure 2. (**C**) The RecA−RecA interaction zone is enlarged to show the residues (represented using the same color code as in (B)) involved in the interaction. Hydrogen bonds and salt bridges are represented by dashed lines, and distances between residues are indicated in Å. ^RecA^F230 is in apolar contact with ^RecA^Q207 of the other subunit.

The N-terminal region of RecA (M1-E39 in *^Ec^*RecA) has been shown to interact with the neighboring monomer to form the nucleoprotein filament, both in absence or in presence of ssDNA and ATP (7). Using Y2H, we confirmed that this region is important for the self-interaction of *^Sp^*RecA (Figure 3). Removing the entire domain (Δ50) abolishes RecA self-interaction when monitored by Y2H, as well as removing the 28 first residues (Δ28), as previously described (14). RecA–RecA interaction is only observable in the context of the full-length protein with this technique (Figure 3A). However, we found that the purified ^Δ28^RecA retains the ability to polymerize, as controlled by SEC-MALS (Supplementary Results and Supplementary Figure S5), although to a lesser extent than ^WT^RecA (data not shown).

**Figure 4.**
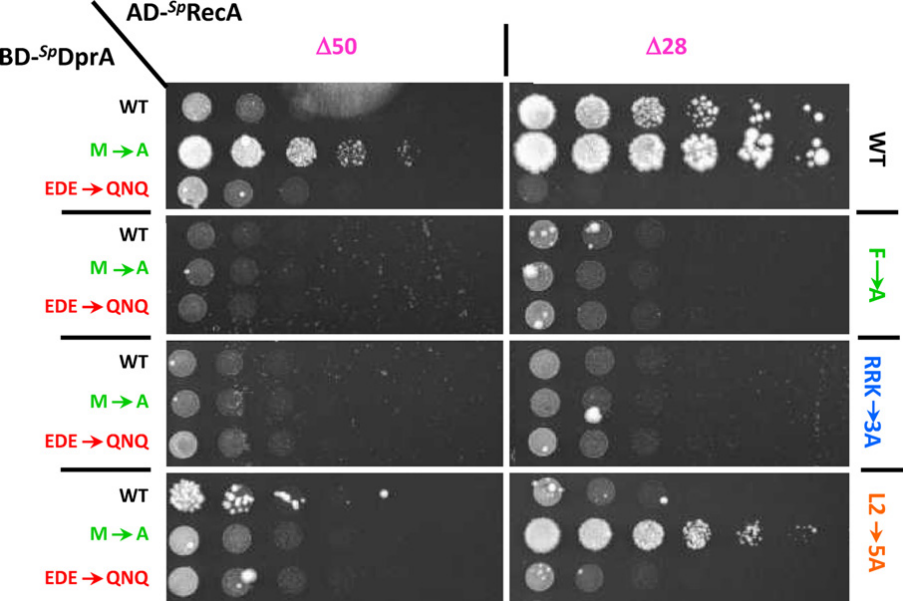
DprA−RecA interaction tests using Y2H assays. Yeasts expressing wild-type or mutant *^Sp^*DprA as Gal4 binding domain fusion (BD-*^Sp^*DprA) and variants of RecA as Gal4 activation domain fusions (AD-*^Sp^*RecA) were spotted as a series of 1/5th dilutions on selective medium lacking histidine. Plates were incubated for 5 days at 28°C. The left column indicates the nature of DprA, the upper line defines the length of RecA and the right column shows its content in terms of mutations.

*A contrario* the full-length RecA does not display any detectable interaction with DprA in Y2H experiments (Supplementary Figure S6). The origin for this lack of interaction might be that full-length RecA preferentially self-associates rendering RecA−DprA interaction undetectable in Y2H. However, DprA−RecA interaction becomes clearly observable in the ^Δ28^RecA context and to a lesser extent in the ^Δ50^RecA context, suggesting additionally that the [28–50] segment of RecA might be involved in the interaction with DprA. As previously noted, this region has been omitted in the docking simulation due to its high mobility. The ^M238A^DprA mutant (described below), actually leading to the strongest interaction between DprA and RecA, follows the same tendency with a higher interaction with ^Δ28^RecA than with ^Δ50^RecA (Figure 4).

### The ^DprA^M238 / ^RecA^F230 positions are pivotal determinants in the DprA−RecA interaction

The major anchor residues in the best HADDOCK/InterEvScore model are the ^DprA^M238 and ^RecA^F230 residues, which are in contact with each other (Figure 2). According to the crystal structure of *^Sp^*DprA, the ^DprA^M238 is at the center of the EDE triad and is thus likely involved in the interface. The strictly conserved ^RecA^F230 is fully exposed at the surface of RecA monomer and is part of a large apolar patch involved in the oligomerization of RecA. In the model, ^RecA^F230 makes hydrophobic contacts with an apolar patch on DprA involving ^DprA^M238, ^DprA^L262 and the aliphatic moiety of ^DprA^E265. It is noteworthy that this ^RecA^F230 residue is also prominently involved in other interface models with a different relative orientation of DprA and RecA, including the best ZRANK model (data not shown). In order to challenge the involvement of ^DprA^M238 and ^RecA^F230 in the interaction, both residues were mutated to Ala. Upon mutation of ^RecA^F230, neither ^WT^DprA nor ^M238A^DprA interact with the ^Δ28/F230A^RecA or ^Δ50/F230A^RecA construct (Figure 4). This seems to confirm the involvement of F230 in the interface between DprA and RecA. Surprisingly, the ^M238A^DprA mutant interacts both with ^Δ28^RecA and ^Δ50^RecA with a better efficiency than its wild-type counterpart (Figure 4). A possible molecular interpretation for this phenotype of increased interaction might come from the proximity in space of ^DprA^M238 and ^RecA^F230 observed in the favored interface model (best HADDOCK/InterEvScore model; Figure 2C). The ^DprA^M238A mutant would create a hydrophobic pocket in the interface where ^RecA^F230 might relocate in a more favorable rotameric state, stabilizing the interaction (Figure 2D). The total loss of interaction with the ^F230A^RecA mutant, in all the interaction-matrix experiments, can be interpreted by the fact that this M/F pivot is a major determinant for the interaction. This ^RecA^F230 mutation also strongly reduces the self-interaction of full-length RecA (Figure 3).

### The acidic patch of DprA: face to face with a basic patch on RecA

*^Sp^*DprA and *^Sp^*RecA have very polarized surfaces, electrostatic potentials likely contributing to the DprA−RecA complex binding affinity (Figure 1 and Supplementary Figure S1). Mutation of the EDE triad of DprA (E235/D243/E265) into ^QNQ^DprA leads to a drastic decrease in the interaction with RecA (Figure 4), as previously shown by TAP-tag experiments in *S. pneumoniae* (18). Three basic residues (R189/R226/K267) involved in the RecA multimerization interface form salt bridges with the three acidic residues of the EDE triad in the interface of the best HADDOCK/InterEvScore model (Figure 2), suggesting that they could contribute to the affinity between DprA and RecA. The R189A/R226A/K267A triple mutant (^RRK^RecA) was tested in the Y2H assays. The DprA−RecA interaction was lost in all conditions of the Y2H matrix results (Figure 4), showing the great importance of these electrostatic interactions. These results tend to agree with the preference given to the best HADDOCK/InterEvScore model, where the basic triad plays an important role in the interaction, in contrast with the best ZRANK model that can be ruled out on the basis of this experiment. By contrast, ^RRK^RecA disrupts only partially the self-interaction of RecA in its full-length context (Figure 3A), suggesting that the electrostatic part of the interaction represents only a fraction of the forces involved in the oligomerization of RecA (Figure 3C).

### Contribution of RecA Loop2 in the interaction with DprA

Besides the N-terminal helix of RecA, the ^RecA^F230 anchor and the basic RRK patch, the global orientation of the docking model suggested that still another region of RecA could be potentially involved in the interaction with DprA. It corresponds to the so-called Loop2 that is unresolved in the *apo*-RecA structures (PDB ID: 1U94) (34) but contacts DNA in several crystal structures of *^Ec^*RecA oligomers solved in complex with dsDNA or ssDNA molecules (PDB IDs: 3CMT, 3CMU, 3CMV, 3CMW, 3CMX) (2) (Figure 3B). Loop2, together with another loop, the so-called Loop1, frame the entrance of a groove at the interface between two RecA monomers. This groove contains the nucleotide binding site of one RecA monomer. Interestingly, the basic RRK triad and ^RecA^F230 lie in the vicinity of this groove. A specific motif in Loop2, highly conserved and strongly apolar (V212/G213/V214/M215/F216), prompted us to check whether it may contribute to the DprA−RecA association. A quintuple mutant switching all 5 residues of the VGVMF motif into alanines was constructed and tested in Y2H assay. The quintuple mutant has only a weak effect on *^Sp^*RecA self-interaction (Figure 3A) but significantly decreases the interaction strength between ^WT^DprA or ^M238A^DprA and ^Δ28^RecA or ^Δ50^RecA (Figure 4). Mutant proteins were all expressed at similar levels in yeast cells as demonstrated by western blot (Supplementary Figure S4). These results support a model in which at least three regions of RecA contribute to the interaction with (i) the neighboring ^DprA^acid/^RecA^basic triads and ^DprA^M238/^RecA^F230 anchors, (ii) the ^DprA^dimerization interface/^RecA^N-terminal helix and (iii) the apolar motif of Loop2. The first region could target a region of DprA accessible in the dimeric form while the other two could be required to remodel the DprA dimeric interface and promote the release of ssDNA from DprA.

### Location of the ssDNA binding site on *^Sp^*DprA

**Figure 5.**
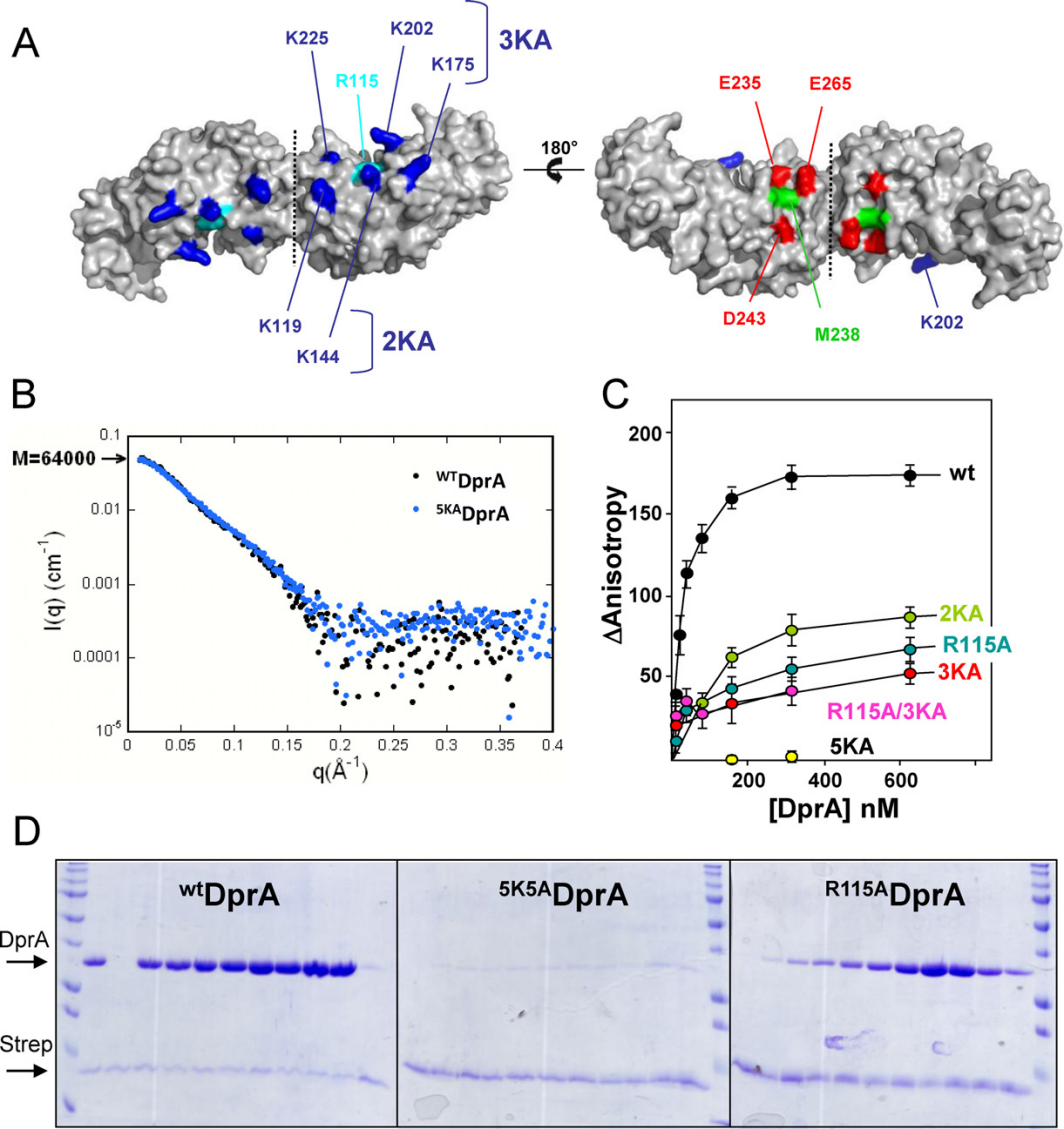
Localization of the DNA binding residues on DprA. (**A**) The electropositive (left) and electronegative (right) faces of the DprA dimer are represented. The five lysines mutated in five alanines to disrupt the interaction with ssDNA were colored in dark blue, and the conserved arginine 115 is in cyan. The acidic EDE triad is in red, and the central M238 is in green. (**B**) SAXS analysis indicates that ^5KA^DprA is a dimer in solution, confirming its good folding. The experimental scattering curve I(q) of ^WT^DprA (black dots; taken from (18)) was superimposed on the curve of ^5KA^DprA (blue dots). (**C**) Equilibrium binding of ^WT^DprA, ^R115A^DprA, ^R115A/3KA^DprA, ^2KA^DprA, ^3KA^DprA and ^5KA^DprA to fluorescein-labeled dT20. (**D**) *In vitro* binding assay between purified ^WT^DprA, ^5KA^DprA and ^R115A^DprA according to increased concentration (20 to 640 pmol), and 80 pmol of 65-mer oligonucleotide bounded to streptavidine (Strep) (see Materials and Methods for details). Proteins are indicated on the left of the 14% SDS-PAGE gel stained by Coomassie-blue.

We previously mentioned that the electrostatic properties and conservation profiles of DprA strongly suggested that the face opposite to the EDE triad (Figure 1A and Figure 5A) was involved in the binding of ssDNA. A recent structure of a complex between DprA from *Helicobacter pylori* (*^Hp^*DprA) and ssDNA (35) confirmed that the positively charged patch conserved at the surface of DprA is likely involved in the binding of ssDNA. The highly conserved R115, whose equivalent R52 was shown to be crucial in the interaction of *^Hp^*DprA with ssDNA, has been targeted. To further probe the binding of ssDNA and DprA, a quintuple mutant targeting the other accessible basic residues K119/K144/K175/K202/K225 on the basic face of DprA has also been constructed (^5KA^DprA), as well as two intermediate mutants in which we removed two and three of the charges, respectively (^2KA^DprA and ^3KA^DprA; see Figure 5A and Supplementary Materials and Methods). Two different techniques were used to monitor the binding of ssDNA to these mutants: spectrofluorimetry using small oligonucleotides as in (18) and pull-down titration on magnetic beads coated with a 65-mer oligonucleotide. Both methods showed that the single mutant ^R115A^DprA has a significant decrease in affinity for ssDNA with respect to ^WT^DprA (Figure 5C and D). However, mutations of the other five basic residues in the ^5KA^DprA mutant resulted in a much stronger loss of interaction showing that R115 alone is not sufficient for binding ssDNA oligonucleotides. We controlled by SAXS that ^5KA^DprA remains dimeric (Figure 5B). For the partial charge deletion mutants, ^2KA^DprA and ^3KA^DprA mutants, we observed a decrease in ssDNA binding similar to that of the ^R115A^DprA mutant (Figure 5C). No further decrease was observed in the combined ^R115A/3KA^DprA mutant suggesting that ^3KA^DprA mutant is defective enough to suppress the contribution of R115 to the binding. The residual binding of ^3KA^DprA with respect to ^5KA^DprA is thus only due to the lysines mutated in the ^2KA^DprA mutant and not due to the presence of R115. Altogether, these results show that contacts between *^Sp^*DprA and the ssDNA involve several basic residues and consequently that the entire patch is needed for a stable interaction.

## DISCUSSION

**Figure 6.**
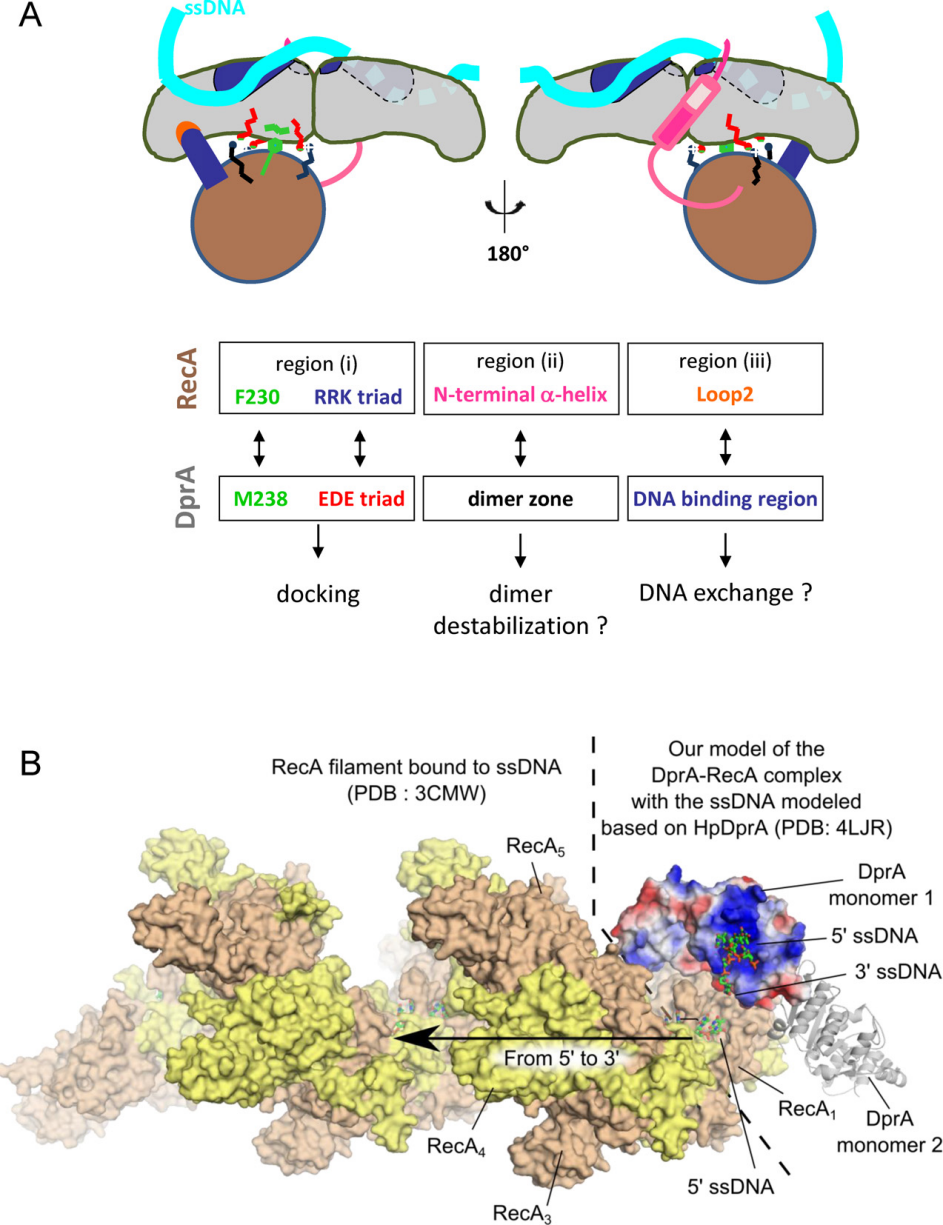
(**A**) Mechanistic model proposition. Schematic representation of the favored HADDOCK-best InterEvScore model, summarizing the functional residues and zones studied. (**B**) Global structural model for the assembly between DprA, RecA and ssDNA integrating the structural information provided by two other studies: (a) the first monomer in the structure of the *^Ec^*RecA filament bound to ssDNA (PDB: 3CMW (2)) was superimposed with the structure of the *^Sp^*RecA model assembled with the *^Sp^*DprA dimer as in the best HADDOCK/InterEvScore model. (b) The location of the ssDNA complexed to *^Sp^*DprA was approximately modeled by superimposing the structure of *^Hp^*DprA (PDB: 4LJR) on the first monomer of *^Sp^*DprA (PDB: 3UQZ).

Combining docking simulations with extensive mutagenesis experiments, we provide novel insights into the architecture of the DprA−RecA complex that lead to a plausible mechanism driving *S. pneumoniae* DprA and RecA association. Unlike *^Bs^*DprA (16), it has been previously suggested that *^Sp^*DprA binds to the protein-free transforming ssDNA and nucleates *^Ec^*RecA onto ssDNA (14), implying a direct interaction between DprA and RecA. TAP-tag experiments, Y2H and electron microscopy experiments previously showed that the two proteins (as well as the monomeric DprA^AR^ mutant) are able to interact, but without providing evidence on whether or not an intermediate partner is needed (14,18). Here, we have demonstrated by pull-down experiments that the two purified proteins interact by using the ^Δ28^RecA mutant. In this experiment, RecA is a mix of monomers and dimers, according to SEC-MALS data (see Supplementary Results and Supplementary Figures S2 and S5). We thus confirm that DprA and RecA from *S. pneumoniae* can interact directly, without the necessity for a third partner. We postulate that the DprA dimer in complex with ssDNA offers a platform for the nucleation of the RecA−ssDNA filament by forming a stable binding site for the first monomer of RecA via direct interaction between DprA and RecA (Figure 6).

The major outcome of our study is that regions which are involved in the self-association of both partners also appear as directly involved in the formation of the DprA−RecA complex. Guided by the most likely and consensual docking model, we proposed that three distinct regions in RecA could be involved in the association with DprA (Figure 6A): (i) basic residues (RRK triad) involved in the interface of the self-assembled RecA globular domain, (ii) part of the N-terminal helix located upstream of the globular core domain of RecA, principally via the [28–50] segment, and (iii) the hydrophobic tip of RecA Loop2 contacting ssDNA in the nucleoprotein filament. The interaction of a RecA monomer with a dimer of DprA could be at least partly mediated by region (i) encompassing ^RecA^F230 surrounded by ^RecA^(R189/R226/K267) (Figures 2 and 4). As a dimer, DprA would interact through the ^DprA^M238 residue and the ^DprA^(E235/D243/E265) triad.

In the absence of ssDNA substrate, RecA molecules likely form mixtures of monomeric and oligomeric assemblies (7) that can self-assemble through the same regions as those involved in the catalytically active nucleoprotein filament. We have observed this dynamics of polymerization for *^Sp^*RecA using the SEC-MALS technique (Supplementary Results and Figure S5). In case of RecA oligomers, regions (i) and (ii) are inaccessible to the binding by DprA except for the very first monomer of RecA at the filament end. Our data support a model in which a first RecA monomer is loaded onto ssDNA by DprA-ssDNA, and that this helps to nucleate formation of a RecA-ssDNA filament. Following this hypothesis, loading of ssDNA in RecA filament would occur in the 5′→3′ direction, fully consistent with the preferred directionality of RecA loading as illustrated in Figure 6B (3,36).

At this stage, our model does not need to invoke any of the two non-globular (ii) and (iii) regions of RecA to account for the binding between RecA and DprA. However, our previous study (18) suggested that the structure of DprA as a symmetric tail-to-tail dimer has to be significantly remodeled to transfer ssDNA from DprA to RecA. Our hypothesis supported by Y2H data is that regions (ii) and (iii) rather could play a role in the destabilization/disruption of DprA dimer interface and in the transfer process, probably necessitating some flexibility of these two regions, to allow an effective DprA−RecA interaction. Their strong hydrophobic character might contribute to their ability to perturb the stable DprA homodimer. The DprA monomer has only a 2.6-fold weaker binding affinity for dT20 compared to the DprA dimer, but it has specifically lost its ability to form aggregates with dT90 oligonucleotides as shown previously by gel retardation (18). Here, we provide an initial analysis of the binding site of *^Sp^*DprA for the ssDNA. The totally conserved R115 is involved in the binding, in agreement with the recently published X-ray structure of *^Hp^*DprA complexed with dT35 ssDNA, which involves R52, the equivalent of R115 in *H. pylori* (35). On the other hand, we have characterized a basic patch of five residues (K119/K144/K175/K202/K225) in the neighborhood of R115 that is crucial for the interaction. Only three of the charged patch residues, including the K202 (corresponding to the *^Hp^*K137), are conserved in *^Hp^*DprA (Supplementary Figure S7). The 5KA mutant abolishes the interaction, while the partial patch mutations 2KA and 3KA are only affected at the same level as the R115A single mutant (Figure 5). In our docking model, this patch is localized on the opposite side with respect to the RecA interaction site, close to the Loop2 of RecA (region iii, Figure 6B and Supplementary Figure S7C). We propose that Loop2, dedicated to the binding of RecA on ssDNA, might compete with the basic patch of DprA to facilitate the transfer of ssDNA from DprA to RecA. If the destabilization of the DprA dimer in favor of a monomer is a functional step in this process, as suggested in (18), we can postulate that the disappearance or at least the release of the network of aggregates can help to load RecA. This would provide a molecular explanation for the transfer of ssDNA from DprA to RecA, with regard to the possible contribution of the N-terminal helix of RecA (region ii) to destabilize the DprA dimer (Figure 6A).

It was previously shown that the DprA-like Smf from *B. subtilis* (*Bs*) and full-length *^Bs^*RecA interact, but not *^Sp^*DprA and full-length *^Sp^*RecA (14). However, *^Sp^*RecA lacking the first 28 residues interacts with *^Sp^*DprA (14). Here we have shown that the apparent lack of interaction between full-length *^Sp^*RecA and *^Sp^*DprA was not due to a weak interaction between both proteins when analyzed in the yeast two-hybrid assay because ^M238A^DprA enhances this interaction. Heterologous interactions were also documented, since *^Sp^*DprA interacts with full-length *^Bs^*RecA and *^Bs^*Smf with full-length *^Sp^*RecA, suggesting that conserved residues might be involved in these protein–protein interactions. It has also been shown that *^Sp^*DprA can recruit *^Ec^*RecA (14), inferring that an interaction between the heterologous proteins may exist. However, the acidic EDE triad is not highly conserved among the DprA family. To further investigate this issue we have systematically analyzed the surface of experimental (*S. pneumoniae*, *Rhodopseudomonas palustris* and the recent *H. pylori*) and homology based models of DprA from a representative set of species (Supplementary Figure S1). We observe the presence of an acidic patch located in a region similar to the one defined for *S. pneumoniae* in the eight transformable model species analyzed over the whole bacterial tree. We always find at least two acidic residues (most often three) that are structurally located in a position very close to their counterpart in *S. pneumoniae* (listed in Supplementary Table S1). These positions are not always strictly aligned in the multiple sequence alignment, but this is consistent with the high versatility of electrostatic interactions observed on a large-scale analysis of homologous interfaces (37). For instance, in the crystal structure of *R. palustris* DprA, the acidic residue corresponding to E235 is located one helix turn downstream at a position equivalent to 239 in *S. pneumonia*. Moreover, we also observe on the surface of the models of all eight species the presence of an apolar patch close to the acidic patch. This apolar patch might accommodate the RecA residue corresponding to F230 (Supplementary Figure S1). Altogether, these observations suggest that similar regions of DprA (in the vicinity of the acid triads/dyads and of the apolar patches) may bind RecA (by contacting the conserved basic residues together with the aromatic residue corresponding to *^Sp^*RecA F230) in a manner similar to that obtained through the docking simulation.

The global model arising from these analyses is that first, globular domains would dock to each other involving region (i) of RecA, while non-globular regions such as (ii) and (iii) could either strengthen this interaction or contribute to destabilize DprA−ssDNA complex either directly through the Loop2 or by interacting further with the dimeric interface of DprA (Figure 6).

## SUPPLEMENTARY DATA

Supplementary Data are available at NAR Online.

SUPPLEMENTARY DATA
