# Supplementary material for: Molecular determinants of the DprA−RecA interaction for nucleation on ssDNA
Source: Nucleic Acids Res. 2014 Apr 29;42(11):7395–408. doi: 10.1093/nar/gku349 (PMC4066776; doi:10.1093/nar/gku349)
Supplement: SUPPLEMENTARY DATA [file supp_42_11_7395__index.html]

Molecular determinants of the DprA−RecA interaction for nucleation on ssDNA — Molecular determinants of the DprA−RecA interaction for nucleation on ssDNA — SUPPLEMENTARY DATA 

# Molecular determinants of the DprA−RecA interaction for nucleation on ssDNA

## SUPPLEMENTARY DATA

**Files in this Data Supplement:**

- SUPPLEMENTARY DATA
