# Supplementary material for: Molecular determinants of the DprA−RecA interaction for nucleation on ssDNA
Source: Nucleic Acids Res. 2014 Apr 29;42(11):7395–408. doi: 10.1093/nar/gku349 (PMC4066776; doi:10.1093/nar/gku349)
Supplement: SUPPLEMENTARY DATA [file supp_gku349_nar-02860-v-2013-File008.pdf]

SUPPLEMENTARY INFORMATION

SUPLEMENTARY MATERIALS AND METHODS

Phylogenetic and structural analysis of DprA homologs.

174 sequences of DprA orthologs extracted from the PhylomeDB (1) were aligned using the Mafft algorithm with the einsi protocol (2). The resulting alignment was trimmed to remove the regions generating gaps in more than half of the sequences of the alignment. From this alignment, the PhyML algorithm (3) was used to infer a phylogenetic tree shown in Supplementary Figure S1 with standard parameters (LG substitution model, 4 substitution rate categories, NNI protocol for tree improvement). The structural models of DprA homologs represented in this figure were obtained using the beta SwissModel server (4). Electrostatic surface was generated using PyMOL (The PyMOL Molecular Graphics System, Version 1.5.0.4 Schrödinger, LLC) while the apolar patch was calculated using our in-house protocol as detailed in (5). Approximate position of the ssDNA was simply modelled by superimposing the structures of ^{Hp}DprA complexed to ssDNA (PDB: 4LJR).

Cloning, overproduction, purification of DprA mutants for DNA interaction disruption and of $^{\Delta 28}$RecA

The quintuple K119A/K144A/K175A/K202A/K225A mutant of DprA (^{5KA}DprA), harbouring an His-tag at the C-terminus, was produced using a cDNA synthesized by GenScript with additional *Nco*I and *Xho*I restriction sites for subcloning in the pET28 vector. The double K119A/K144A (^{2KA}DprA) and triple K175A/K202A/K225A (^{3KA}DprA) mutants were constructed by exchanging the [*Nco*I-*Nde*I] and [*Nde*I-*Xho*I] fragments respectively in pET28-^{WT}DprA. The ^{R115A}DprA mutant was constructed by site-directed mutagenesis using the QuikChange® II Site-Directed Mutagenesis Kit. The overproduction in BL21(DE3)pLysS and the purification of the proteins were performed as described previously for the wild-type DprA, except that the buffer for purification contains 200 mM NaCl instead of the 2 M required for the stability of the WT protein (6). $^{\Delta 28}$RecA and $^{\Delta 28\text{-}F230A}$RecA were constructed by PCR using pGAD-RecA constructs and subcloned in pET28 with an His-tag at the C-terminus. The purification process includes a Ni-NTA step followed by a size exclusion chromatography as previously described for ^{WT}DprA.

Quality controls by Small-angle X-ray scattering measurements

SAXS experiment with the ^{5KA}DprA was performed using the Nanostar instrument at IBBMC in Orsay. Data were collected as described previously for the ^{WT}DprA (6), at protein concentrations of 1 and 5 mg/mL, in 50 mM MES pH 6.5, 2M NaCl.

Monitoring the oligomeric state of $^{\Delta 28}$RecA by SEC-MALS

Size-exclusion chromatography coupled to multi-angle laser light scattering (SEC-MALS) was used to determine the oligomeric state of $^{\Delta28}$RecA according to the concentration. Sample injection, chromatography, and detection were carried out using a UV detector (PDA 2600, Malvern) and a Triple Detector Array (TDA302) system (Malvern) coupled in line to a GPCmax chromatographic system (Malvern). 2 samples of 200 µl of RecA (1 and 2 mg/mL) and one sample of 80 µL of RecA (4 mg/mL) were successively injected at a flow rate of 0.5 ml/min on a Superdex 200 HR 10/30 column (GE Healthcare) equilibrated in buffer 200 mM NaCl, 20 mM Tris-HCl pH 7.5. Elution was followed by a UV-visible spectrophotometer, a differential refractometer, a 7° low angle light scattering detector (LALS), a 90° right angle light scattering detector (RALS), and a differential pressure viscometer. The data were collected and processed with the program OmniSEC (Viscotek). Molecular weight was directly calculated by combining the absolute light scattering and the refractive index measurements. Concentration calculation of the sample from the refractive index signal was extracted by using the *dn*/*dc* value (0.185) of the BSA as a standard.

DNA binding assays

The fluorescence anisotropy titration was measured in triplicates in a Fluoromax-4 (Horiba Scientific) spectrofluorometer, at 20 ℃, in a final reaction v olume of 200 µL buffered with 50 mM NaCl, 20 mM Hepes pH 6.5, 2.5% (vol/vol) glycerol, and supplemented with 10 nM of a dT15-mer 5'-labeled with fluoresceine (GeneCust). The excitation wavelength was set at 490 nm, and emission was observed at 525 nm (10-nm bandwidth). ^{5KA}DprA injections were 0.25–1 µL from a 10 mg/mL stock solution. The data were treated with SigmaPlot 12.0 (Systat Software).

Pull-down assays of DprA on magnetic beads coated by ssDNA

25 µl of Dynabeads M-280 streptavidin per assay (Invitrogen) were incubated 15 min at 4℃ in 2 M NaCl, 20 mM Tris-HCl pH 7.5, with 80 pmol of athe 65-mer oligonucleotide 5'-CGTCGTTTTACAACGTCGTGACTGGGAAAACCCTGGCGTTACCCAACTTAATCGCCTTGCAGCA-3' biotinilated (with biotin TEG; Genecust). Beads were washed with 200 µl of the same buffer, resuspended in 200 µl of buffer A (200 mM NaCl, 20 mM Tris-HCl pH 7.5) supplemented with 20 to 640 pmol of purified DprA. After 30 min of incubation at 800 rpm and 20℃, beads were washed in 200 µl of buffer A, drained and resuspended in 10 µl of SDS-PAGE loading buffer. The proteins were separated on 14 % SDS-PAGE and revealed by Coomassie blue staining.

Pull-down assays of DprA on magnetic beads coated by His-tagged RecA

5 µl of PureProteome Nickel Mag Beads per assay (Merck Millipore) were incubated with 80 pmol of purified $^{\Delta28}$RecA and incubated in 200 mM NaCl, 20 mM Tris-HCl pH 7.5 (buffer A) 30 min at 20℃ under agitation (800 rpm) in a 96 wells plate in a Thermomixer (Eppendorf). Beads were washed in 200 µl of buffer A and resuspended in 200 µl of the same buffer supplemented with various quantities of purified ^{WT}DprA, ^{QNQ}DprA, ^{5KA}DprA or monomer ^{AR}DprA, as indicated in the Supplementary Figure S2. After 30 min of incubation at 800 rpm and 20℃, b eads were washed in 200 µl of buffer A, drained

and resuspended in 10 µl of SDS-PAGE loading buffer. The proteins were separated on 14 % SDS-PAGE and revealed by Coomassie blue staining.

SUPPLEMENTARY RESULTS

Analysis of RecA solution concentrated at 1 mg/mL (upper panel of Supplementary Figure S5) showed one major peak eluted at 14.77 mL giving a calculated average molecular weight of 52 kDa (theoretical molecular weight of RecA is 43 kDa). This unique peak is not totally symmetrical and is populated with two species of RecA : the first one (around 14 mL) consists of a small amount of the dimeric form (MW around 80 kDa) and the second one (elution after 14.77 mL) represent the majority the protein obtained as a monomer (MW : 45 kDa).

When increasing the concentration of RecA to 2 mg/mL (middle panel of Supplementary Figure S5) the peak eluted at 14.77 mL is not monodisperse and consists of a mix of dimer (90 kDa eluted at 13.4 mL) and monomer (45 kDa eluted at 15.1 mL). This peak represents 64% of the total RecA. A second peak is observed at 11.1 mL and corresponds to a higher degree of oligomerization of RecA (MW around 240 kDa which may be consistent with an hexameric form of the protein).

At 4 mg/mL (Lower panel of Supplementary Figure S5), oligomeric RecA elutes mainly around 10,5 mL (MW around 350 kDa) and in the void volume of the column (8.8 mL) with a small portion behaving as dimer (13.5 mL, MW around 105 kDa) and monomer (15.5 mL; MW around 47 kDa), corresponding to 37% of RecA.

Together, those 3 runs clearly show that RecA starts to oligomerize above 1 mg/mL in a concentration-dependent manner leading to highly ordered oligomeric species at 4 mg/mL.

SUPPLEMENTARY TABLE S1 : Index of the acidic residues in different DprA homologs whose structural location would be equivalent to that of the acidic triad in *S. pneumoniae* (E235, D243,E265) and which contribute to form the acidic patches depicted in Supplementary Figure S1.

**Species**	**Acidic position 1**	**Acidic position 2**	**Acidic position 3**
S. pneumoniae	**E235**	**D243**	**E265**
S. aureus	E243	(E247)	E273
B. subtilis	E245	E249	-
N. meningitidis	E251	E255	D277
V. cholerae	E246	E250	(E286)
R. palustris	D258	E262	D280
D. radiodurans	D263	D289	(E303)
H. pylori	D152	D217	-

SUPPLEMENTARY FIGURES

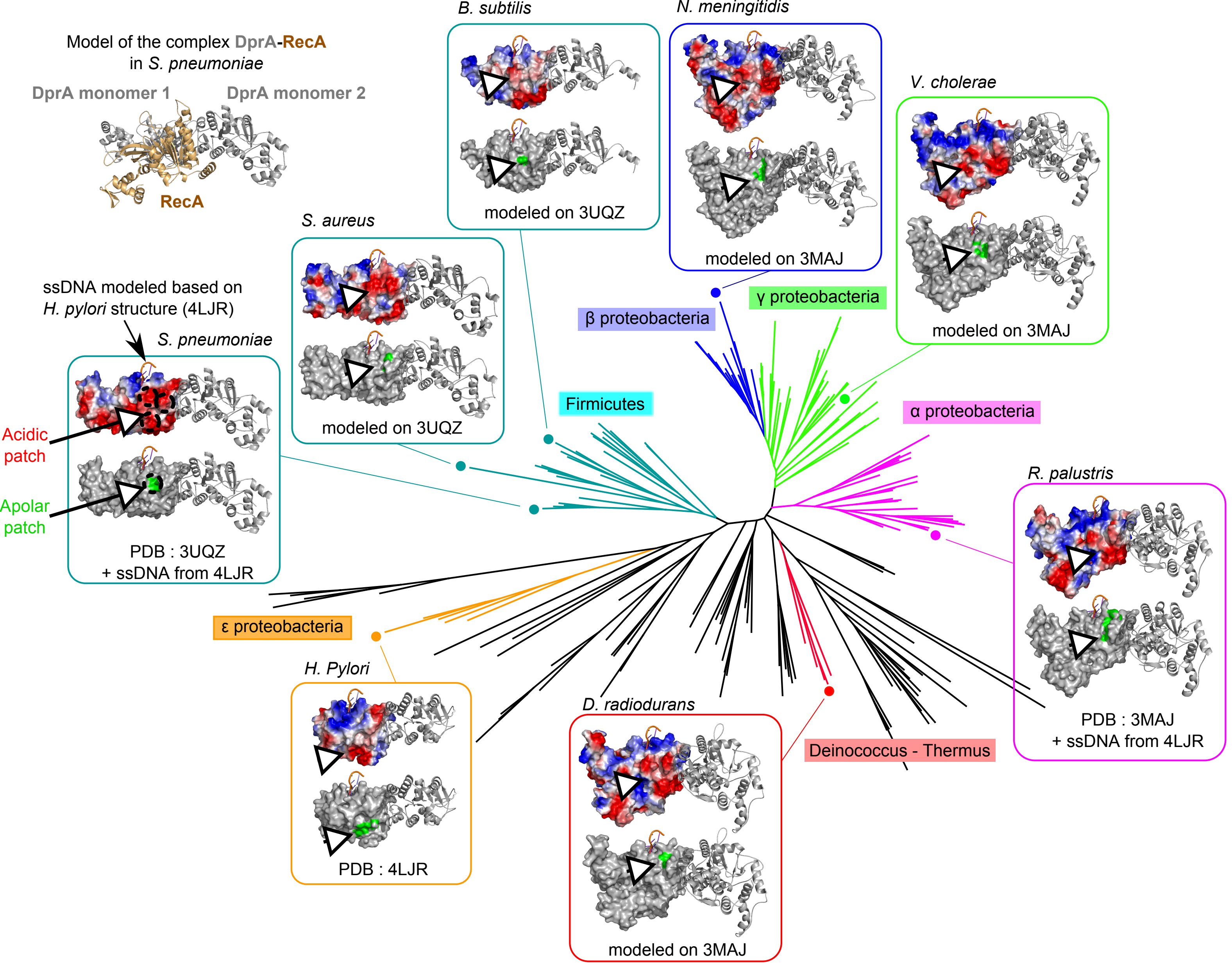

Supplementary Figure S1

Analysis of DprA surface in several bacterial species focused on the region modeled as binding to RecA. A model of the DprA−RecA complex is shown in the upper-left corner of the figure. The central phylogenetic tree was built from a set of 174 orthologues of DprA extracted from the PhylomeDB (1) and the tree branches were colored following species categories. For 8 transformable model species reported in squared panels, the X-ray structure of DprA dimer (*S. pneumoniae, R. palustris, H. pylori*) or a model derived from these structures is represented in the same orientation as in the model of the complex. In every panel, a surface and cartoon representations of the first and second monomer are displayed, respectively. Electrostatic properties, calculated from a simple coulombic law, are mapped on the surface of the upper structure while the location of the apolar patch closest to the position of the acidic triad is shown in green at the surface of the lower structure. In the S. pneumoniae panel, the positions of the acidic triad and of the apolar patch centred on M238 are indicated by dotted circles and in the other panels the corresponding locations are indicated by two white arrows. The location of the ssDNA was modelled by homology, based on the structure of HpDprA complexed with ssDNA (PDB: 4LJR).

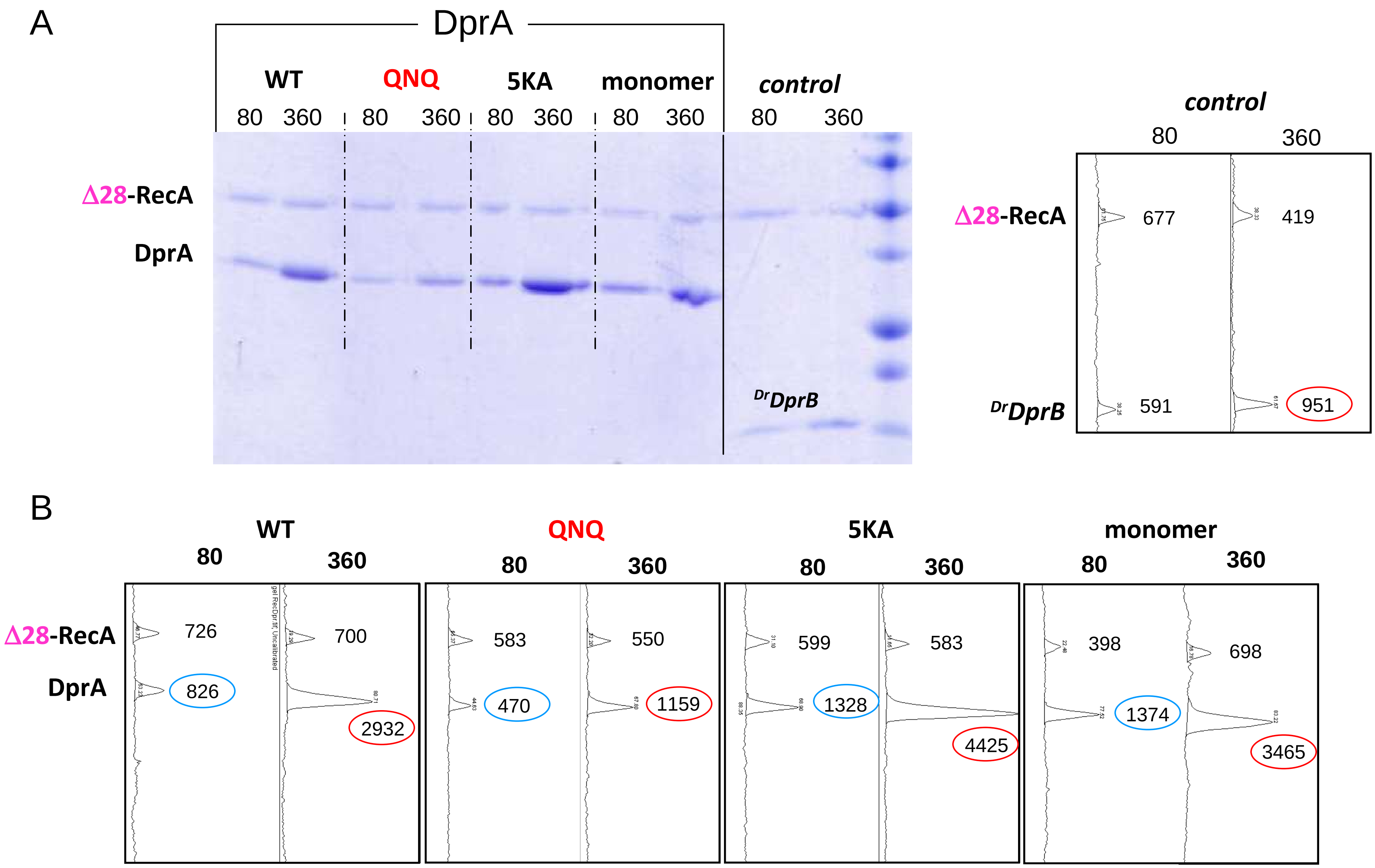

Supplementary Figure S2 Direct DprA−RecA interaction. (**A**) *In vitro* binding assay between purified WTDprA, QNQDprA, 5KADprA, ARDprA (monomer) or DprB from *D. radiodurans* as a control, at 80 and 360 pmol, and 80 pmol of D28RecA (see Supplementary Materials and Methods for details). Proteins are indicated on the left of the 14 % SDS-PAGE gel stained by Coomassie-blue. (**B**) Profile plot of the SDS-PAGE using ImageJ software.

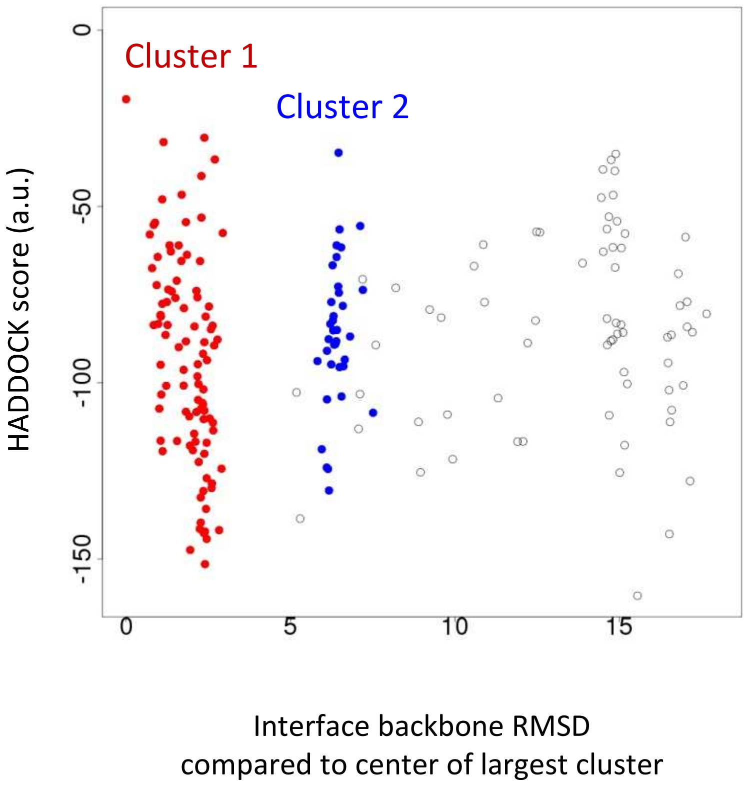

Supplementary Figure S3

Representation of the results of the HADDOCK run for the DprA−RecA interface. The HADDOCK score (arbitrary units) is represented as a function of the interface backbone rmsd (Å) of all solutions with respect to the center of the largest cluster (Cluster 1). The “best HADDOCK model” corresponds to a consensus model from Cluster 1, while the “second-best HADDOCK model” corresponds to a consensus model from Cluster 2.

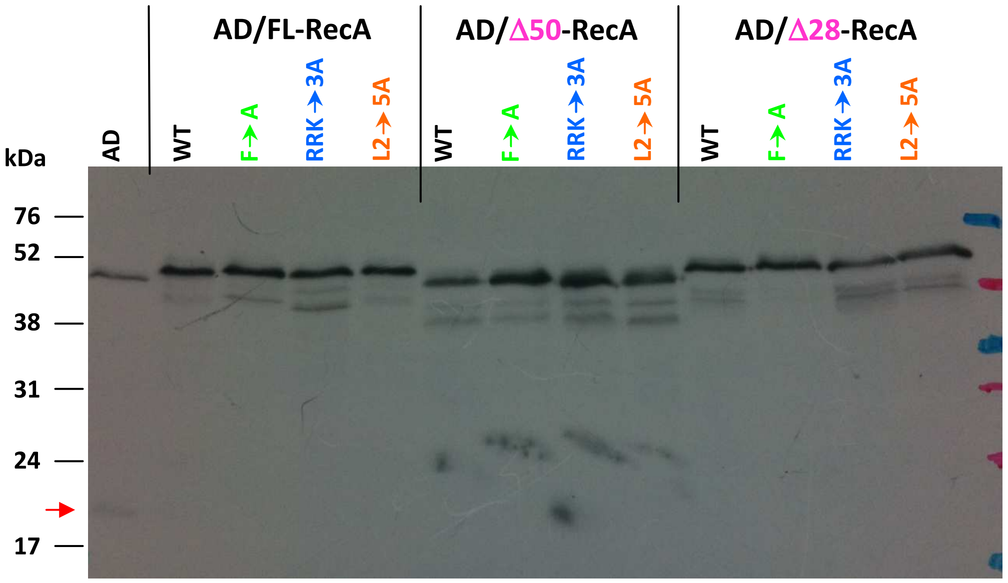

Supplementary Figure S4

DprA–RecA interaction tests using Y2H assays: control of protein expression level in yeast. The various constructions of *Sp*RecA (including the full-length versions of the Supplementary Figure S2) fused to the activation domain of Gal4 were revealed by SDS-PAGE followed by Western blotting using anti-Gal4-AD antibodies.

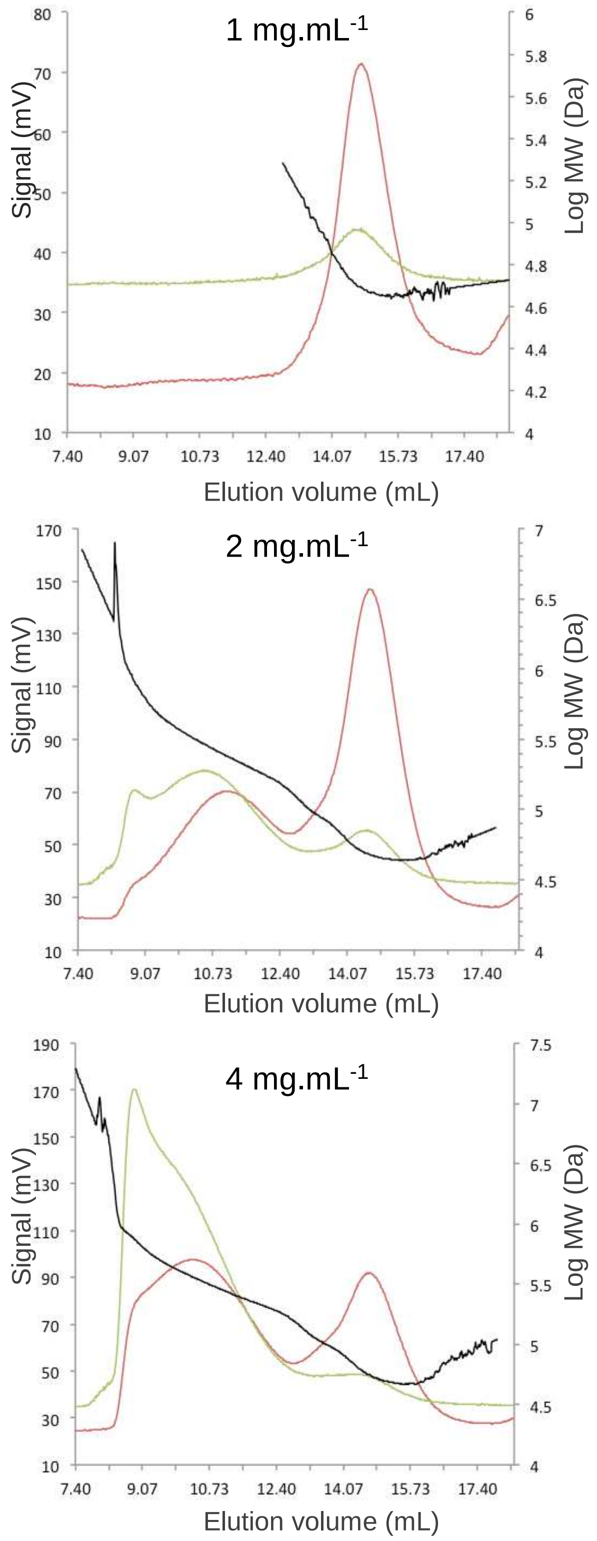

Supplementary Figure S5

RecA polymerization analysis by SEC-MALS. Chromatograms of 3 RecA solutions loaded at different concentrations are shown: 1 mg/mL (upper panel), 2 mg/mL (middle panel) and 4 mg/mL (lower panel). Intensity of the signal (mV) of the refractive index (red line) and Right Angle Light Scattering (Green line) is shown on the left Y-axis. Calculation of the molecular weight (Da) of the different monomeric and oligomeric species of RecA (black line) is shown on the right Y--axis.

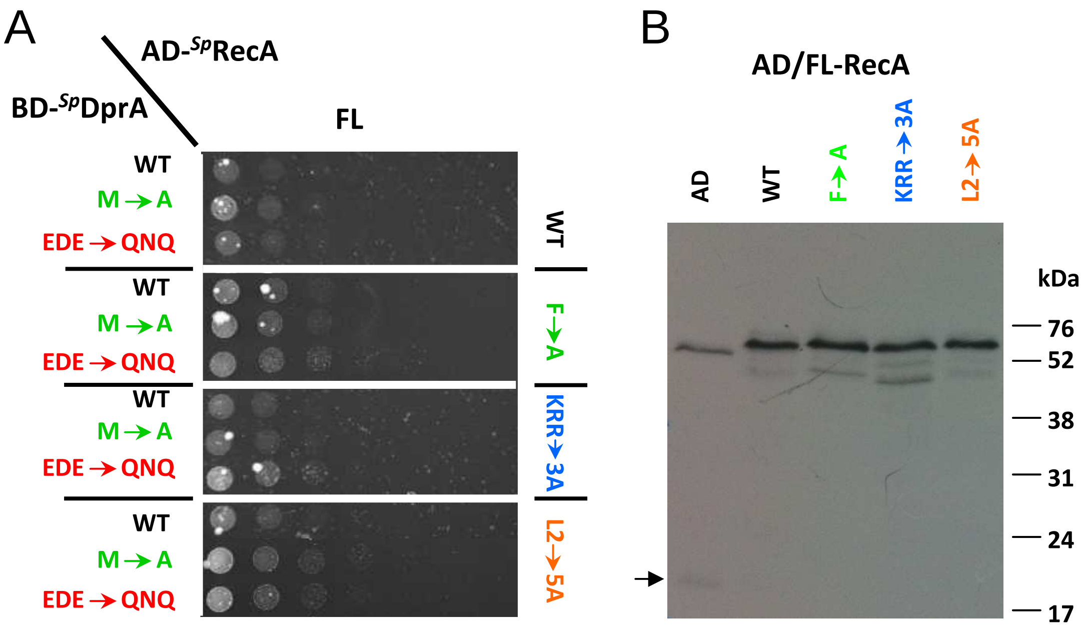

Supplementary Figure S6

RecA polymerization (**A**) Full-length DprA−RecA interaction tests using Y2H assays. Yeasts expressing wild-type or mutant *Sp*DprA as Gal4 binding domain fusion (BD-*Sp*DprA) and variants of RecA as Gal4 activation domain fusions were spotted as a series of 1/5th dilutions on selective medium lacking histidine. Plates were incubated for 5 days at 28℃. The left column indic ates the nature of DprA, the upper line represents the full-length RecA and the right column shows its content in terms of mutations. (**B**) Control of protein expression level in yeast. The various constructions of the full-length *Sp*RecA fused to the activation domain of Gal4 were revealed by SDS-PAGE followed by Western blotting using anti-Gal4-AD antibodies.

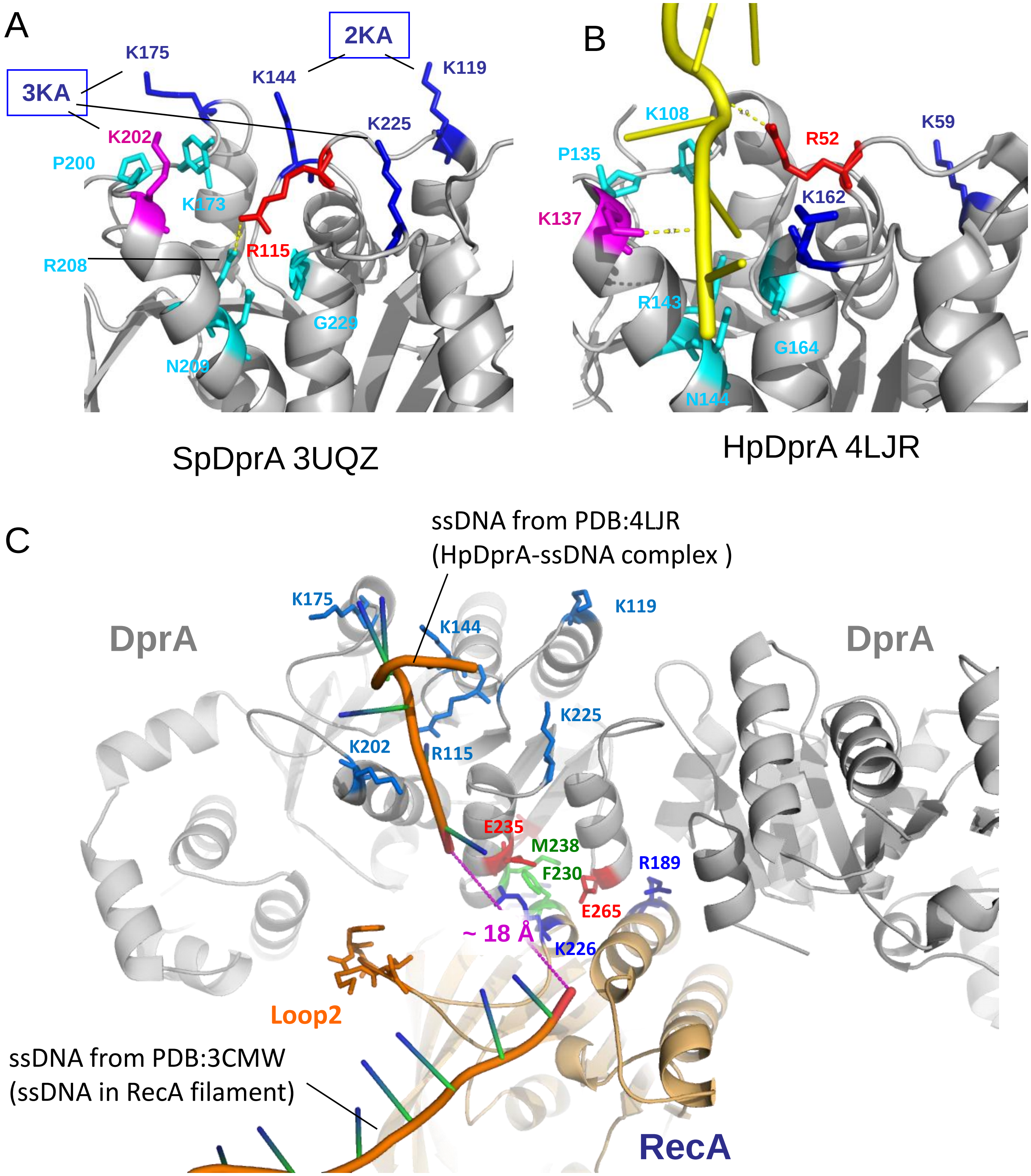

Supplementary Figure S7

Location of DprA residues involved in the interaction with ssDNA. (**A**) Ribbon view with *Sp*DprA residues (from 3UQZ). (**B**) Ribbon view with *Hp*DprA residues (from 4LJR). (**C**) Details of the structural model for the assembly between DprA, RecA and ssDNA, according to Figure 6B.

SUPPLEMENTARY REFERENCES

1. Huerta-Cepas, J., Capella-Gutierrez, S., Pryszcz, L.P., Marcet-Houben, M. and Gabaldon, T. (2014) PhylomeDB v4: zooming into the plurality of evolutionary histories of a genome. *Nucleic acids research*, **42**, D897-902.
2. Katoh, K. and Standley, D.M. (2013) MAFFT multiple sequence alignment software version 7: improvements in performance and usability. *Molecular biology and evolution*, **30**, 772-780.
3. Guindon, S., Dufayard, J.F., Lefort, V., Anisimova, M., Hordijk, W. and Gascuel, O. (2010) New algorithms and methods to estimate maximum-likelihood phylogenies: assessing the performance of PhyML 3.0. *Systematic biology*, **59**, 307-321.
4. Kiefer, F., Arnold, K., Kunzli, M., Bordoli, L. and Schwede, T. (2009) The SWISS-MODEL Repository and associated resources. *Nucleic acids research*, **37**, D387-392.
5. Andreani, J., Faure, G. and Guerois, R. (2012) Versatility and invariance in the evolution of homologous heteromeric interfaces. *PLoS computational biology*, **8**, e1002677.
6. Quevillon-Cheruel, S., Campo, N., Mirouze, N., Mortier-Barriere, I., Brooks, M.A., Boudes, M., Durand, D., Soulet, A.L., Lisboa, J., Noirot, P. *et al.* (2012) Structure-function analysis of pneumococcal DprA protein reveals that dimerization is crucial for loading RecA recombinase onto DNA during transformation. *Proc Natl Acad Sci U S A*, **109**, E2466-2475.
